# Induction therapy with paclitaxel and bevacizumab followed by switch maintenance therapy with eribulin in Japanese patients with HER2-negative metastatic breast cancer: a multicenter, collaborative, open-label, phase II clinical study for the SBCCSG 35 investigators

**DOI:** 10.1186/s12885-018-4556-6

**Published:** 2018-06-20

**Authors:** Kenichi Inoue, Jun Ninomiya, Tsuyoshi Saito, Kei Kimizuka, Masafumi Kurosumi

**Affiliations:** 1 Division of Breast Oncology, Saitama Cancer Center, 780 Komuro, Ina-machi, Kita-adachi-gun, Saitama, 362-0806 Japan; 2 Department of Breast Surgery, Ninomiya Hospital, 2-22-23 Shinei, Soka-shi, Saitama, 340-0056 Japan; 3 Department of Breast Surgery, Japanese Red Cross Saitama Hospital, 1-5 Shintoshin, Chuo-ku, Saitama-shi, Saitama, 330-8553 Japan; 4 Department of Breast Surgery, Kasukabe Medical Center, 6-7-1 Chuo, Kasukabe-shi, Saitama, Japan; 5 Department of Pathology, Saitama Cancer Center, 780 Komuro, Ina-machi, Kita-adachi-gun, Saitama, 362-0806 Japan

**Keywords:** Metastatic breast cancer, Eribulin, Paclitaxel, Bevacizumab, Switch maintenance therapy, Metastasis

## Abstract

**Background:**

To examine the efficacy and safety of induction therapy with paclitaxel and bevacizumab followed by switch maintenance therapy with eribulin (ISMT) in Japanese patients with HER2-negative metastatic breast cancer (MBC).

**Methods:**

Patients, who had previously undergone a maximum of 2 regimens of chemotherapy, received 3 cycles of induction therapy with paclitaxel (90 mg/m^2^ intravenously on days 1, 8, and 15 followed by 1-week drug holiday) and bevacizumab (10 mg/kg intravenously after the completion of paclitaxel administration on days 1 and 15). Patients who had complete response, partial response, or stable disease underwent switch maintenance therapy with eribulin (1.4 mg/m^2^ intravenously on days 1 and 8 followed by 1-week drug holiday). The primary endpoint was time to treatment failure (TTF) for ISMT.

**Results:**

Fifty-one eligible patients (median age: 66 years; range: 35–74) were enrolled: 19 (37.3%) and 32 (62.7%) had stage IV and recurrence, respectively, 42 (82.4%) had visceral metastases, and 45 (88.2%) received eribulin—38 of whom showed disease progression, and 40 (78.4%) underwent post therapy. Median TTF was 9.2 months (95% confidence interval [CI]: 7.3–11.1), median progression-free survival was 10.7 months (95% CI: 9.6–11.8), and median overall survival was 20.0 months (95% CI: 16.0–24.0). Relative dose intensity was 97.7% (range: 33.3–100.0) for induction therapy and was 83.3% (range: 49.3–100.6%) for eribulin maintenance therapy. The most common adverse event was alopecia (51 [100%]) in induction therapy and was peripheral sensory neuropathy (37 [82.2%]) in eribulin maintenance therapy. Eribulin was effective with manageable tolerability.

**Conclusions:**

ISMT may be a promising therapeutic option for patients with MBC.

**Trial registration:**

UMIN000015971. Registration date: January 1, 2015.

## Background

Approximately 90% of patients diagnosed with breast cancer undergo surgery for the treatment of their primary malignancy [1], and up to 5% of patients present with distal metastatic breast cancer (MBC) at the time of diagnosis [2]. Breast cancer recurs in about 40% of patients who underwent surgery [1], the residual recurrence-free survival rates at 5 and 10 years after adjuvant and neoadjuvant systemic therapy are 89 and 80%, respectively [3], and the median survival of patients with MBC is 28 months [4]. Therefore, MBC is often incurable despite modern treatments [5]. The goals of treatment are the alleviation of symptoms, extension of survival, and improvement in the quality of life (QOL) of patients [6].

Anthracycline (A)- and taxane (T)-containing regimens (collectively, AT therapy) are preferred chemotherapeutic modalities for patients with MBC [7, 8]. Treatments other than AT therapy are frequently selected when breast cancer progressed or recurred after AT therapy was conducted as pre- or postoperative chemotherapy. Nevertheless, chemotherapy after recurrence still remains to be improved.

Eribulin, a nontaxane microtubule dynamics inhibitor that is the first agent in the halichondrion class, has the following unique mechanisms of action among tubulin-targeting compounds: 1) inhibition of microtubule polymerization without depolymerization induction and 2) induction of nonproductive tubulin aggregate formation [9–11]. Eribulin monotherapy has shown efficacy and safety in patients with MBC who had received anthracyclines and/or taxanes [12–14] and in Japanese counterparts [15, 16].

On the other hand, the E2100 Study [17] that is a pivotal study of combined therapy using paclitaxel and bevacizumab (the PB regimen) in patients with human epidermal growth factor receptor 2 (HER2)-negative MBC demonstrated the efficacy and safety of the regimen as first-line chemotherapy for the study population, and its independent blind review [18] validated them; however, the incidence of grade 3/4 peripheral neuropathy was as high as 23.6%. An open-label phase II study of bevacizumab in combination with weekly paclitaxel in Japanese patients with MBC [19] suggested that the activity and tolerability of the combination therapy as first-line therapy were reproducible in the Japanese patient population; again, the incidence of peripheral neuropathy was high. Our previous cohort study [20] also verified both the high efficacy and safety of the PB regimen but disclosed a high dose reduction rate of 28% in patients who had received the initial paclitaxel dose of 90 mg/m^2^. Hence, a need to develop a chemotherapeutic regimen that is less likely to cause dose reductions in paclitaxel emerged. Peripheral neuropathy lowers the QOL of patients with MBC who are undergoing AT therapy and may cause dose reductions or treatment discontinuation. The reported incidences of peripheral neuropathy have been higher for taxane-based therapy [17, 21, 22] than for eribulin monotherapy [12–16]. The rational of switching from the former to the latter emerged in expectation of the potential reduction in peripheral neuropathy by eribulin [16, 20].

Under the abovementioned clinical circumstances, we conducted induction therapy with paclitaxel and bevacizumab followed by switch maintenance therapy (ISMT) with eribulin alone in expectation that this therapeutic paradigm would more favorably maintain the QOL of patients with MBC than does AT therapy. The present first prospective study on ISMT with eribulin alone intended to examine the efficacy and safety of the paradigm in Japanese patients with MBC.

## Methods

### Study design

The present multicenter, collaborative, open-label, Phase II clinical study of ISMT (SBCCSG 35; University Hospital Medical Information Network identifier: 000015971) was conducted in Japanese patients with MBC. Patients were recruited centrally. All patients provided written informed consent before enrollment. The study protocol was approved by the Institutional or Central Ethics Committee, and the study was conducted in accordance with the Declaration of Helsinki, Good Clinical Practice, and local ethical and legal regulations.

The primary endpoint for ISMT was time to treatment failure (TTF). The secondary endpoints for ISMT were progression-free survival (PFS), the overall response rate (ORR), overall survival (OS), safety, peripheral neuropathy, and the assessment of QOL.

### Patients

At the time of entry, female patients were checked for age, height, body weight, history, complications, HER2/neu expression, hormone status, hematology, and blood chemistry.

Patients were eligible if they met the following requirements: 1) a female diagnosed with histologically or cytologically confirmed breast cancer; 2) a definite diagnosis of MBC; 3) 20 to 74 years of age; 4) Eastern Cooperative Oncology Group (ECOG) performance status, 0 to 2; 5) measurable lesions according to the response evaluation criteria in solid tumors (RECIST) [23]; 6) major organs of well-conserved functions (bone marrow, liver, kidney, and lungs), i.e., neutrophil count: ≥ 1500/μL, platelet count: 80,000/μL, hemoglobin: ≥ 9.0 g/dL, aspartate aminotransferase (AST), alanine aminotransferase (ALT), total bilirubin, and serum creatinine: ≤ 2.5-fold the upper limit at the site; 7) grade ≤ 1 peripheral neuropathy at screening; 8) HER2-negative breast cancer; 9) an expected survival period of ≥ 3 months since the day of administration onset; 10) no clinical concerns about electrocardiograms; 11) no history of treatment with paclitaxel, bevacizumab, or eribulin after metastasis; and 12) written informed consent provided by the patient herself. The key exclusion criteria were as follows: systemic infection involving fever (≥ 38.0°C), history of hypersensitivity to investigational drugs and their solvents, metastasis to the brain requiring treatment, interstitial pneumonia, pulmonary fibrosis, poorly controlled hypertension or diabetes mellitus, active double cancer, history of mental impairment, central nervous system impairment, or cerebrovascular neuropathy, pregnancy, breast-feeding, or women of childbearing age, and patients whom the investigator or subinvestigator considered ineligible.

Patients, who achieved disease control, underwent ISMT until disease progression, development of intolerable toxicities, consent withdrawal, or investigator’s discretion to discontinue study treatment. Patients, who showed progressive disease (PD), underwent post therapy.

### Treatment

Patients received 3 cycles of induction therapy with paclitaxel (90 mg/m^2^ intravenously, once daily, on days 1, 8, and 15 followed by 1-week drug holiday) and bevacizumab (10 mg/kg intravenously after the completion of paclitaxel administration on days 1 and 15) as PB therapy, followed by 1-week drug holiday on day 22. In patients whose status was rated to be stable disease (SD) or better during cycle 3 of the PB regimen, switch maintenance therapy with eribulin 1.4 mg/m^2^ alone was conducted for 2 consecutive weeks during which they underwent infusion, once weekly, on days 1 and 8, as well as 1-week drug holiday at week 3. This cycle was repeated to the extent possible.

The following anticancer therapies were prohibited during the study period because of their potential effects on the assessment of the present clinical study: hormone therapy, immunotherapy, chemotherapy, radiotherapy, surgery, and systemic steroid therapy.

### Assessments

#### Efficacy

Tumor lesions were monitored at screening, within 10 weeks (± 3 weeks) after the date of first administration, and every 6 weeks (± 3 weeks) after the date of previous monitoring of the lesions thereafter. The date of monitoring could be rescheduled as needed when a deterioration in disease status was suspected. Tumor response was assessed according to Response Evaluation Criteria in Solid Tumors version 1.1 [23]. Best overall response was determined based on the following criteria: complete response (CR), partial response (PR), PD, SD, and not evaluable (NE). TTF was defined as the time from enrollment to treatment discontinuation in ISMT due to any causes including disease deterioration, treatment-related adverse events (AEs), and death. PFS was defined as the time from enrollment to an event (death or deterioration, whichever earlier) in ISMT. Overall survival (OS) was defined as the time from enrollment to any confirmed all-cause death in ISMT.

#### Safety

AEs were graded according to the Common Terminology Criteria for Adverse Events, Japanese version 4.0 [24]. AEs of the worst grade were recorded. Peripheral neuropathy was assessed for its time of development and severity, and the QOL of patients in ISMT was evaluated according to the Functional Assessment of Cancer Therapy/Gynecologic Oncology Group-Neurotoxicity (FACT/GOG-Ntx) questionnaire [25].

#### Statistical analyses

The present study was subjected to the per-protocol analysis. Continuous variables are expressed as mean ± SD or counts and percentage, while categorical variables as median and interquartile range. The Kaplan-Meier estimates (median with 95% CI) were calculated for TTF, PFS, and OS. Descriptive statistics were calculated to assess safety data. Patients were analyzed on an intent-to-treat basis, and those who received at least one investigational drug were analyzed for safety.

In our prior clinical study [20], median TTF for the PB regimen was 6.2 months (95% CI: 4.2–8.3). Therefore, we established an expected TTF of 9.0 months and a threshold TTF of 6.2 months in patients with MBC whose response to treatment is SD or better after 3 cycles (2.8 months) of the PB regimen. During the entry and follow-up periods of 24 months and 12 months, respectively, 51 patients will endow the study with 80% power at an α level of 0.05. The target number of patients was set to 53 in consideration of patients who might be excluded from analysis due to ineligibility. A two-tailed *p*-value of < 0.05 was considered statistically significant. Wilcoxon’s signed rank test was conducted to test differences between the pre- and post-treatment scores of the FACT/GOG-Ntx questionnaire. All statistical analyses were made using SPSS version 19 (IBM, Armonk, NY).

## Results

### Patient population

Between January 23, 2015, and February 25, 2016, a total of 53 Japanese female patients with MBC were recruited to the present clinical study at 4 medical institutions in Japan. Two of these patients were ineligible (one patient underwent 4 regimens of chemotherapy and another had systemic infection involving fever of 38°C). Therefore, 51 were enrolled in the study; 19 (37.3%) and 32 (62.7%) had stage IV and recurrence, respectively. Patient characteristics at baseline are shown in Table 1. The number of patients who underwent first-line chemotherapy was 34; and the number of patients who underwent second-line chemotherapy was 17, of whom 6 and 11 received anthracycline and oral 5-fluorouracil, respectively. None of them received both classes of anticancer drugs (Table 1). Among them, 43 (84.3%) were postmenopausal, 32 (62.7%) had MBC, 42 (82.4%) had visceral metastases, 16 (31.4%) had 5 or more metastases, 27 (53.0%) underwent hormone therapy previously, as well ass 42 (82.4%), 38 (74.5%), and 9 (17.6%) had estrogen receptor (ER)-positive, progesterone receptor (PgR)-positive, and triple-negative breast cancer, respectively. The most predominant site of metastases was the liver, followed by the lung and local lesion. Fifty-one and 45 patients were assessable for the efficacy and safety of the PB regimen and eribulin maintenance therapy, respectively.

**Table 1:** Demographic and clinical characteristics of patients at baseline (*N* = 51)

Characteristic	n (%)
Age, years	
Median	66
Range	35–74
ECOG performance status	
0	31 (60.8)
1	10 (19.6)
2	10 (19.6)
Tumor status	
Stage IV	19 (37.3)
Recurrence	32 (62.7)
Status of menopause	
Premenopause	8 (15.7)
Postmenopause	43 (84.3)
Histopathological types	
Common	49 (96.1)
Special	2 (3.9)
Hormone receptor status	
ER-positive	42 (82.4)
ER-negative	9 (17.6)
PgR-positive	38 (74.5)
PgR-negative	13 (25.5)
Triple-negative (ER, PgR, HER2)	9 (17.6)
Pre- or postoperative treatment	
Anthracycline	16 (31.4)
Paclitaxel	4 (7.8)
Docetaxel	12 (23.5)
Hormone therapy	27 (52.9)
Hormone therapy after recurrence	36 (70.6)
First-line chemotherapy	34 (66.7)
Second-line chemotherapy	
Anthracycline	6 (11.8)
Oral 5-fluorouracil	11 (21.5)
Number of metastases	
1	7 (13.7)
2	10 (19.6)
3	13 (25.5)
4	5 (9.8)
5	10 (19.6)
6–8	6 (11.8)
Dominant site of metastases	
Visceral	42 (82.4)
Nonvisceral	9 (17.6)
Site of metastasis	
Bone	35 (68.6)
Liver	32 (62.7)
Local lymph node	27 (52.9)
Lung	21 (41.2)
Local lesion	17 (33.3)
Distal lymph node	15 (29.4)
Pleural effusion	10 (19.6)
Pleura	6 (11.8)
Brain	2 (3.9)
Contralateral mamma	2 (3.9)
Cardiac effusion	1 (2.0)
Spleen	1 (2.0)
Pulmonary lymphangitis	1 (2.0)
Skin	1 (2.0)
Adrenal	1 (2.0)

*ECOG* Eastern Cooperative Oncology Group, *ER* estrogen receptor, *PgR* progesterone receptor, *HER2* human epidermal growth factor receptor 2

### Study drug exposure

Fifty-one and 45 patients in the PB regimen and eribulin maintenance therapy received the investigational drugs, respectively; the median of cycles delivered in the latter was 10 cycles (range: 1–29 cycles). The relative dose intensity (RDI) in the former was 97.7% (range: 33.3–100.0), with 3 dose reductions, 17 dose delays, and 9 dose discontinuations. In contrast, the RDI in the latter was 83.3% (49.3–100.6%), with 16 dose reductions, 35 dose delays, 38 dose discontinuations, and 2 combinations with pegylated granulocyte colony-stimulating factor.

### Efficacy of induction therapy followed by switch maintenance therapy

**Fig. 1:**
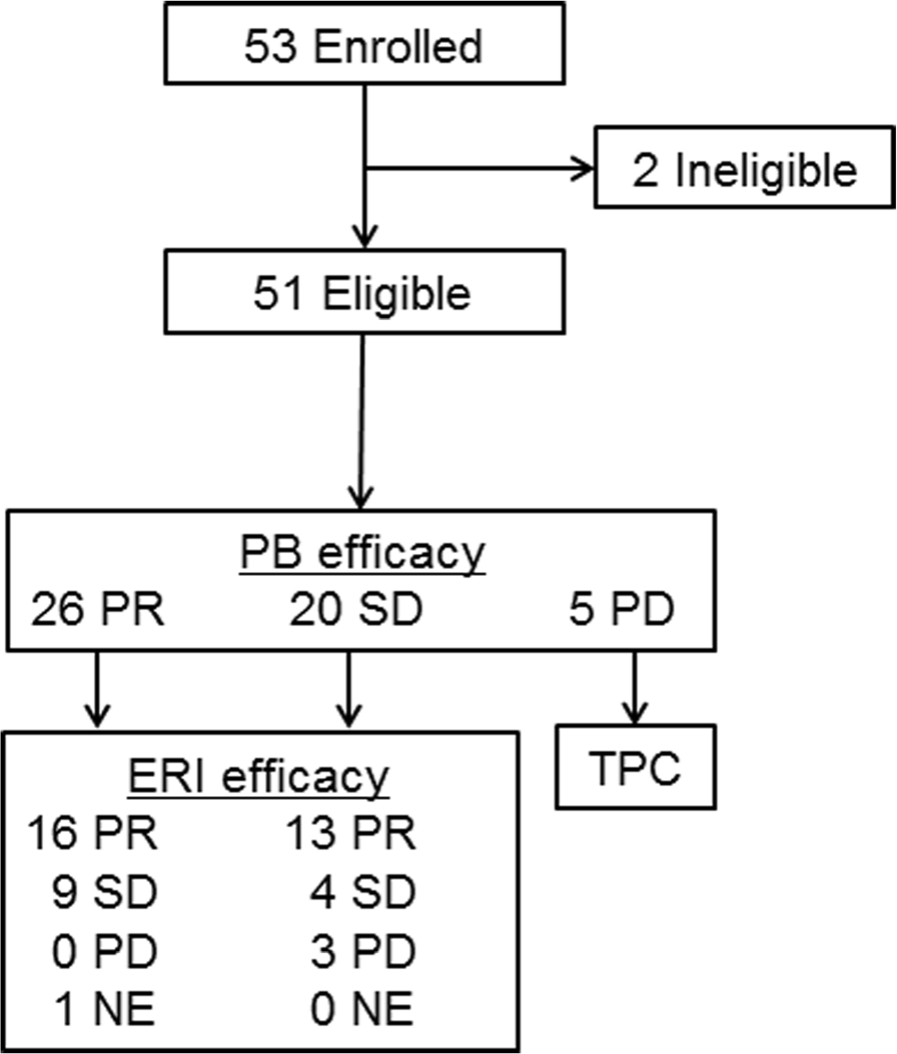
Flow diagram showing best overall responses to ISMT, ISMT, induction therapy followed by switch maintenance therapy, PB, paclitaxel and bevacizumab; PD, progressive disease; PR, partial response; SD, stable disease; ERI, eribulin; NE, not evaluable due to interstitial pneumonia; TPC, treatment of physician’s choice

**Fig. 2:**
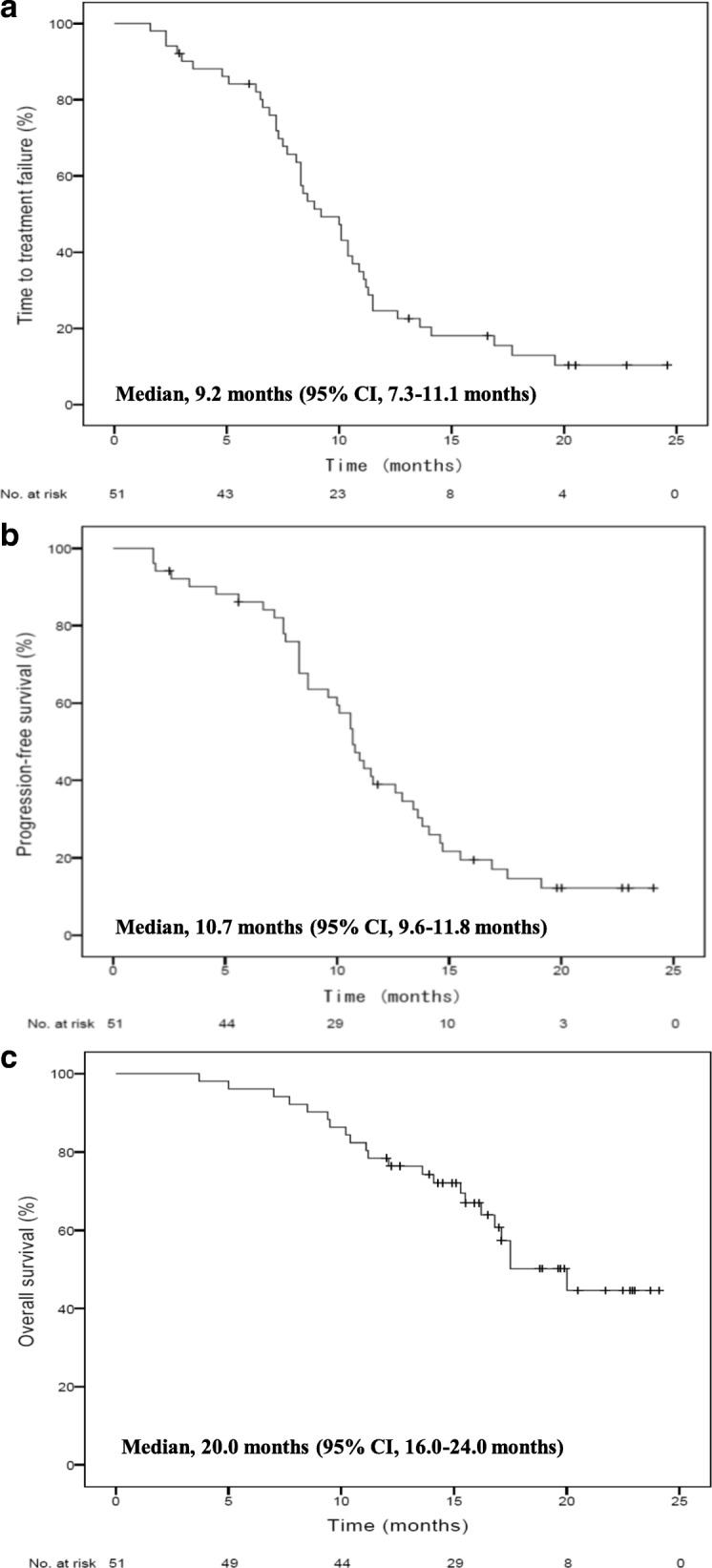
Kaplan-Meier curves for TTF (**a**), PFS (**b**), and OS (**c**) in ISMT with paclitaxel, bevacizumab, and eribulin, ISMT, induction therapy followed by switch maintenance therapy; TTF, time to treatment failure, PFS, progression-free survival, and OS, overall survival

**Fig. 3:**
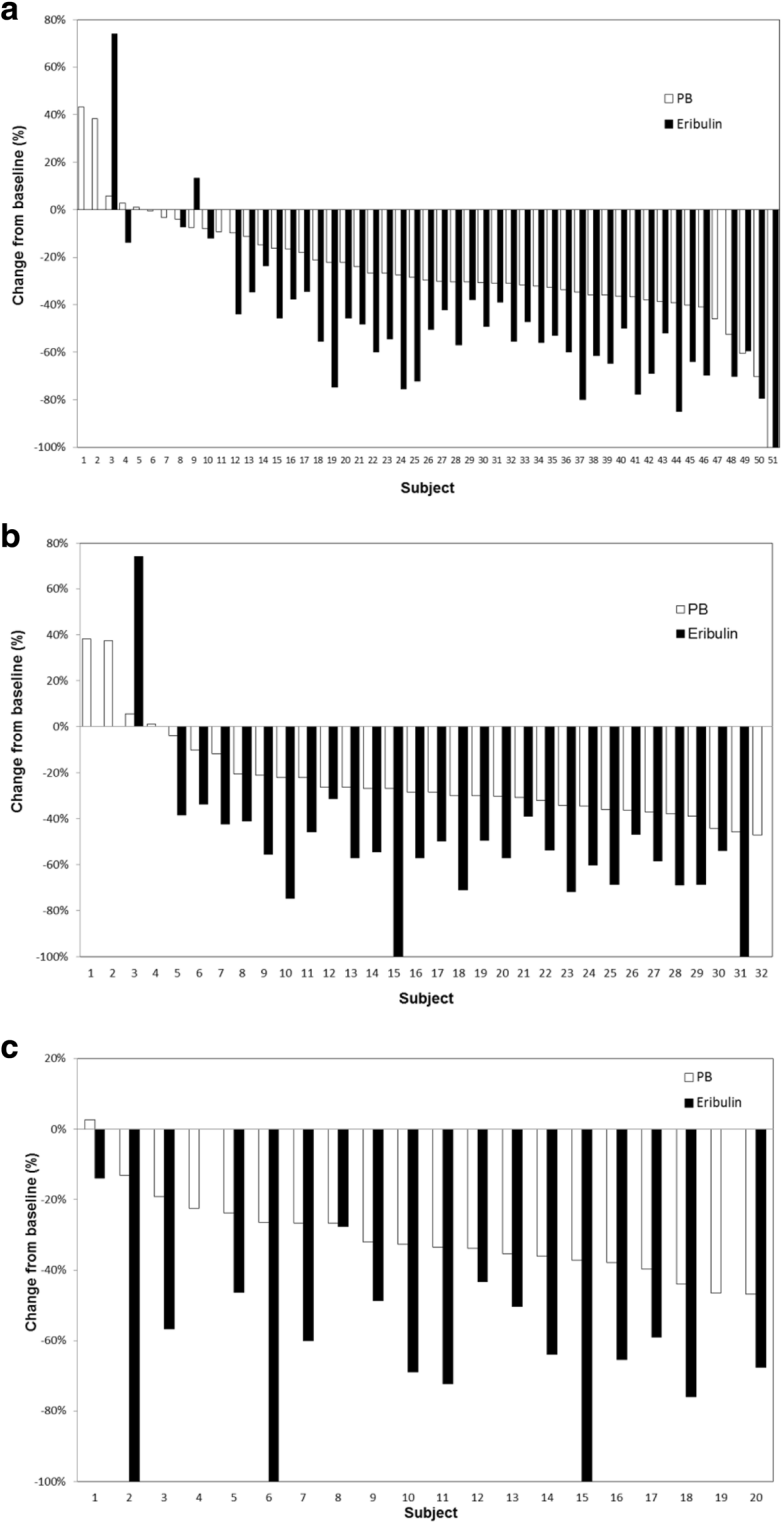
Waterfall plots of percent changes in metastatic tumor size (total sum of the longest single dimension for measurable target lesions) from baseline to the maximal tumor shrinkage by organ regarding best overall response; (**a**), overall; (**b**), liver; (**c**), lung, PB, paclitaxel and bevacizumab

Among 51 enrolled patients, 45 (88.2%) underwent maintenance treatment with eribulin alone—38 of whom had disease progression during a median follow-up of 17 months and 7 did not. The following best response rates were obtained (Table 2), and a flow diagram showing best overall responses to ISMT is shown (Fig. 1). In the PB regimen, the ORR was 51.0% (95% CI: 36.6–65.3) and the disease control rate (DCR: CR + PR + SD) was 90.2% (95% CI: 78.6–96.7). In eribulin maintenance therapy, the ORR was 64.4% (95% CI: 48.8–78.1) and the DCR was 93.3% (95% CI: 81.7–98.6). Furthermore, the following best overall responses were obtained with respect to 17 patients who underwent second-line chemotherapy: for 6 patients who received anthracycline, 3 each were rated to PR and SD; and 11 patients who received oral 5-fluorouracil, PR, SD, and PD were 8, 2, and 1, respectively. The Kaplan-Meier curves for TTF, PFS, and OS in ISMT are shown in Fig. 2. Median TTF was 9.2 months (95% CI: 7.3–11.1) (Fig. 2a), median PFS was 10.7 months (95% CI: 9.6–11.8) (Fig. 2b), and median OS was 20.0 months (95% CI: 16.0–24.0) (Fig. 2c). Waterfall plots (Fig. 3a-c) indicate percent changes in metastatic tumor size (total sum of the longest single dimension for measurable target lesions) from baseline to the maximal tumor shrinkage. In the vast majority of patients with MBC whose best tumor response was rated to be SD or better, consequently, tumor shrinkage was greater for eribulin maintenance therapy than for the PB regimen with respect to the following principal organs that showed the elevated incidences of visceral metastases: overall (Fig. 3a), liver (Fig. 3b), and lung (Fig. 3c). The shrinkage rates in the overall, liver, lung, and soft tissue were 93.3% (42/45), 96.4% (27/28), and 100.0% (18/18), respectively.

**Table 2:** Best overall responses

	Number	Percent
PB regimen (n = 51)		
CR	0	0.0
PR	26	51.0
SD	20	39.2
PD	5	9.8
NE	0	0.0
ORR (CR + PR)	26	51.0
95% CI	36.6–65.3	
DCR (CR + PR + SD)	46	90.2
95% CI	78.6–96.7	
Maintenance treatment with eribulin (*n* = 45)		
CR	0	0.0
PR	29	64.4
SD	13	28.9
PD	3	6.7
ORR (CR + PR)	29	64.4
95% CI	48.8–78.1	
DCR (CR + PR + SD)	42	93.3
95% CI	81.7–98.6	

*BP* bevacizumab + paclitaxel, *CR* complete response, *PR* partial response, *SD* stable disease, *PD* progressive disease, *NE* not evaluable, *ORR* overall response rate, *DCR* disease control rate, *CI* confidence interval

Among 38 patients who had disease progression, 10 had new lesions (6 in the central nervous system and 1 each in the liver, bone, peritoneum, and distal lymph nodes) and 28 had the deterioration of existing lesions.

Forty patients (78.4%), who showed disease progression in ISMT, underwent post therapy; 23 of these patients again underwent the PB regimen (Table 3). The DCR was 75.0% (15/20) for the PB regimen.

**Table 3:** Post therapy

	n	PR	L-SD	SD	PD	NE	PD^a^
ADM/EPI + CPA	3				3		1
S1	3			3			1
PTX + BEV	23	5	1	9	5	3	
ERI	3	1			1	1	2
DTX	1				1		
EXE + EVE	1				1		
CPA	1				1		
None	1						
Pembrolizumab	1					1	
Rx to the whole brain	3	1		1	1		

^a^: Progressive disease in induction therapy

*ADM* doxorubicin, *EPI* epirubicin, *CPA* cyclophosphamide, *PTX* paclitaxel, *BEV* bevacizumab, *ERI* eribulin, *DTX* docetaxel, *EXE* exemestane, *EVE* everolimus, *Rx* radiation, *PR* partial response, *L-SD* long-lasting stable disease, *SD* stable disease, *PD* progressive disease, *NE* not evaluable

Regarding the QOL of patients, the scores of the FACT/GOG-Ntx questionnaire lowered significantly (*p* < 0.001) from the baseline score of 39.6 ± 4.8 to 31.7 ± 8.9 at the end of the PB regimen and to 30.6 ± 9.0 at week 6 of eribulin maintenance therapy. However, no significant difference was found between the PB regimen and eribulin maintenance therapy.

### Safety profile

All of 51 patients who underwent the PB regimen and more than 80% of patients who underwent eribulin maintenance therapy experienced AEs (Table 4). All the AEs were clinically manageable by dose modifications (dose interruptions/delays or dose reductions) or symptomatic treatment. The most common hematologic AE was anemia in the PB regimen and eribulin maintenance therapy, being found in 58.8% (30/51) and 57.8% (26/45) of patients, respectively. Among nonhematologic AEs, alopecia (100%, 51/51) and all-grade peripheral sensory neuropathy (82.2%, 37/45) were most common in patients who underwent the PB regimen and eribulin maintenance therapy, respectively. The grade of peripheral sensory neuropathy deteriorated and improved in 14 and 3 patients who underwent eribulin maintenance therapy, respectively. Grade 3 peripheral sensory and motor neuropathies in the PB regimen and eribulin monotherapy were 15.7 and 37.8%, respectively. Grade 4, hematologic or nonhematologic AEs did not develop. No patient died in ISMT.

**Table 4:** Most common adverse events by CTCAE grade (incidence of all grades: ≥ 15%)

Adverse events	Severity, n (%)			
	All grades	Grade 1	Grade 2	Grade 3
Hematologic				
PB regimen (n = 51)				
Anemia	30 (58.8)	18 (35.3)	9 (17.6)	3 (5.9)
Leukopenia	21 (41.2)	10 (19.6)	8 (15.7)	3 (5.9)
Neutropenia	19 (37.3)	2 (3.9)	9 (17.6)	8 (15.7)
Eribulin maintenance therapy (n = 45)				
Anemia	26 (57.8)	21 (46.7)	4 (8.9)	1 (2.2)
Leukopenia	24 (53.3)	9 (20.0)	10 (22.2)	5 (11.1)
Neutropenia	23 (51.1)	2 (4.4)	12 (26.7)	9 (20.0)
Nonhematologic				
PB regimen (n = 51)				
Alopecia	51 (100.0)	2 (3.9)	49 (96.1)	–
Peripheral sensory neuropathy	37 (72.5)	28 (54.9)	8 (15.7)	1 (2.0)
Increased AST	15 (29.4)	13 (25.5)	1 (2.0)	1 (2.0)
Dysgeusia	15 (29.4)	15 (29.4)	0 (0.0)	–
Epistaxis	15 (29.4)	15 (29.4)	0 (0.0)	0 (0.0)
Fatigue	12 (23.5)	10 (19.6)	2 (3.9)	0 (0.0)
Arthralgia	12 (23.5)	12 (23.5)	0 (0.0)	0 (0.0)
Rash	12 (23.5)	12 (23.5)	0 (0.0)	0 (0.0)
Increased ALT	10 (19.6)	8 (15.7)	2 (3.9)	0 (0.0)
Anorexia	10 (19.6)	7 (13.7)	3 (5.9)	0 (0.0)
Nail discoloration	10 (19.6)	10 (19.6)	–	–
Peripheral motor neuropathy	9 (17.6)	8 (15.7)	0 (0.0)	0 (0.0)
Myalgia	8 (15.7)	8 (15.7)	0 (0.0)	0 (0.0)
Eribulin maintenance therapy (n = 45)				
Peripheral sensory neuropathy	37 (82.2)	18 (40.0)	17 (37.8)	2 (4.4)
Increased AST	25 (55.6)	21 (46.7)	3 (6.7)	1 (2.2)
Dysgeusia	16 (35.6)	13 (28.9)	3 (6.7)	–
Increased ALT	16 (35.6)	14 (31.1)	2 (4.4)	0 (0.0)
Alopecia	15 (33.3)	8 (17.8)	7 (15.6)	–
Fatigue	14 (31.1)	10 (22.2)	4 (8.9)	0 (0.0)
Anorexia	14 (31.1)	11 (24.4)	3 (6.7)	0 (0.0)
Peripheral motor neuropathy	13 (28.9)	10 (22.2)	3 (6.7)	0 (0.0)
Increased creatinine	13 (28.9)	12 (26.7)	1 (2.2)	0 (0.0)
Nausea	11 (24.4)	11 (24.4)	0 (0.0)	0 (0.0)
Arthralgia	10 (22.2)	9 (20.0)	1 (2.2)	0 (0.0)
Myalgia	9 (20.0)	7 (15.6)	2 (4.4)	0 (0.0)
Nail loss	9 (20.0)	4 (8.9)	5 (11.1)	–
Constipation	8 (17.8)	5 (11.1)	3 (6.7)	0 (0.0)
Epistaxis	7 (15.6)	7 (15.6)	0 (0.0)	0 (0.0)
Nail discoloration	7 (15.6)	7 (15.6)	–	–

*CTCAE* common terminology criteria for adverse events, *PB* paclitaxel + bevacizumab, *AST* aspartate transaminase, *ALT* alanine transaminase; −, no definition

## Discussion

In clinical practice where oncologists treat patients with MBC that progressed or recurred after first-line chemotherapy, there is an increasing demand to develop a novel pharmacotherapeutic paradigm that aims at maximizing primary therapy benefit, improving disease control, and conserving an acceptable QOL. The clinical applications of maintenance therapy (i.e., continuation maintenance and switch maintenance) have evolved in such contexts [26]. A diversity of therapeutic armamentariums for maintenance therapy (e.g., chemotherapy, hormone therapy, and targeted therapy) may be tailored to them. Switch maintenance therapy is the introduction of a new and potentially non-cross-resistant agent at the completion of front-line chemotherapy [27] and is intended to maximize the antitumor efficacy of therapy, to minimize treatment-related toxicities, and to maintain the QOL of individual patients. The first pivotal study using this therapeutic strategy, which examined the impact of the timing of therapy, was presented by Fidias et al. for patients with advanced non-small-cell lung cancer [28].

We conducted eribulin maintenance therapy in patients who achieved disease control with the PB regimen in an attempt to minimize cumulative toxicities and drug resistance associated with the prolonged administration of the same drugs [29] in first-line chemotherapy and to increase the possibility of benefiting from maintenance therapy [30] with eribulin. This therapeutic devisal translated into good median TTF, PFS, and OS, although the study patient population was severely affected by MBC as evidenced by the elevated proportions of patients (82.4%) who had visceral metastases and 4 or more metastases (41.2%) at baseline.

Concretely, median PFS of 10.7 months, and median OS of 20.0 months in the present study are longer than 2 to 7 months in PFS [12–16] and 9 to 13 months in OS [12–16] that were reported in previous clinical studies of eribulin monotherapy in patients with pretreated MBC [12–16]. These clinical outcomes from ISMT were favored by the sequential combination of a highly effective chemotherapy (i.e., the PB regimen) and monochemotherapy that is less likely to provoke peripheral neuropathy (i.e., eribulin monotherapy). The present study successfully met the statistical requirements. Namely, median TTF was 9.2 months (95% confidence interval [CI]: 7.3–11.1), thus successfully exceeded the established threshold time of 6.2 months. Furthermore, eribulin maintenance therapy contributed to the acceptable preservation of the QOL of patients, which was indicated by the absence of statistical significance in the scores of the FACT/GOG-Ntx questionnaire between the PB regimen and eribulin maintenance therapy.

The waterfall plot analysis of maximal changes in metastatic tumor size by organ revealed the absence of increased or unchanged tumor size in more than 80% of patients who underwent ISMT according to the protocol despite switching to treatment with eribulin alone.

The present study on ISMT is the first prospective clinical study on switch maintenance therapy with eribulin alone. To date, a number of studies on maintenance therapy after induction therapy with diversified chemotherapeutic agents have been conducted in pretreated patients with MBC. For example, Gligorov et al. [31] conducted a randomized, open-label, phase 3 clinical study (IMELDA) in patients with HER2-negative MBC, in which first-line docetaxel (75–100 mg/m^2^) and bevacizumab (15 mg/kg) was followed by maintenance bevacizumab (15 mg/kg) alone or in combination with capecitabine (1000 mg/m^2^). Consequently, median PFS was 11.9 months for capecitabine plus bevacizumab and was 4.3 months for bevacizumab alone. Furthermore, median OS was 39.0 months for capecitabine plus bevacizumab and was 23.7 months for bevacizumab only. Thus, the addition of capecitabine to bevacizumab maintenance therapy after taxane-based induction treatment for patients with HER2-negative MBC afforded significant and clinically meaningful improvements in both PFS and OS. Furthermore, Alba et al. [32] conducted a multicenter, randomized, phase III control study in patients with MBC to assess first-line induction chemotherapy with doxorubicin (75 mg/m^2^) and docetaxel (100 mg/m^2^) followed by maintenance therapy consisting of pegylated liposomal doxorubicin (PLD; 40 mg/m^2^) or observation. Consequently, median PFS was 8.4 months for PLD against 5.1 months for observation and median OS was 24.8 months for PLD against 22.0 months for observation. In previous clinical studies on maintenance therapy after first-line induction therapy [30–34], median PFSs ranged between 4 and 12 months and median OSs ranged between 23 and 39 months. Hence, the results from ISMT are comparable to these clinical outcomes despite the severer clinical background of our patient population.

The concept of switching from a chemotherapy to another with greater tolerability emerged as described in the IMELDA study in patients with HER2-negative MBC [31]—“Switching to a more tolerable chemotherapy, such as capecitabine, with a different mechanism of action, while continuing VEGF inhibition, might be a more effective treatment strategy.” In the present study, we intended to examine the efficacy and safety of ISMT in which eribulin was used as switch maintenance therapy instead of capecitabine in the same patient population and found the following: 1) none of patients rated to PR in the PB regimen experienced disease progression; 2) switch maintenance therapy with eribulin alone was well tolerated (i.e., peripheral neuropathy as assessed with the FACT/GOG-Ntx questionnaire did not deteriorate) in consistency with previous clinical studies of eribulin therapy [12–16]; and 3) the incidence of alopecia drastically lowered from 100% with the PB regimen to 33.3% with switch maintenance therapy with eribulin alone.

The present study has several limitations. First, sample size is relatively small, which impedes a robust conclusion equivalent to that is drawn from a large-scale clinical study. However, our study was endowed with sufficient statistical power and is the first to provide valid clinical evidence on eribulin maintenance therapy after the PB regimen. Second, our study is not a randomized controlled clinical study and is therefore of lower evidence level; nevertheless, ISMT is a novel chemotherapeutic paradigm that may be beneficial for patients with MBC who are under critical conditions after tumor progression or recurrence. Third, switch maintenance therapy was conducted with eribulin alone, not in combination with paclitaxel or bevacizumab, in an attempt to precisely assess its pharmacological effects on breast cancer that progressed or recurred after the PB regimen. Consequently, inter-regimen comparisons were precluded for maintenance therapy.

## Conclusion

We present the first prospective clinical study on induction therapy with paclitaxel and bevacizumab that was followed by switch maintenance therapy with eribulin alone in Japanese patients with HER2-negative metastatic breast cancer. This therapeutic regimen is effective, safe, and feasible, and therefore may be considered as a promising therapeutic option for patients with MBC that progressed or recurred after first-line chemotherapy.

## Abbreviations

AEs: adverse events
ALT: alanine aminotransferase
AST: aspartate aminotransferase
AT therapy: anthracycline and taxane therapy
CI: confidence interval
CR: complete response
DCR: disease control rate
ECOG: Eastern Cooperative Oncology Group
ER: estrogen receptor
FACT/GOG-Ntx: Functional Assessment of Cancer Therapy/Gynecologic Oncology Group-Neurotoxicity
HER2: human epidermal growth factor receptor 2
ISMT: induction therapy followed by switch maintenance therapy
IV: intravenous
MBC: metastatic breast cancer
NE: not evaluable
ORR: overall response rate
OS: overall survival
PB regimen: paclitaxel and bevacizumab regimen
PD: progressive disease
PFS: progression-free survival
PgR: progesterone receptor
PLD: pegylated liposomal doxorubicin
PR: progressive disease
QOL: quality of life
RDI: relative dose intensity
SBCCSG: Saitama Breast Cancer Clinical Study Group
SD: stable disease
SD: standard deviation
TTF: time to treatment failure
UMIN: University Hospital Medical Information Network
